# PP44 Cost Effectiveness Of Canal-Wall-Down Mastoidectomy Followed By Hearing Reconstruction With Active Bone Conduction Implant For Patients With Cholesteatoma

**DOI:** 10.1017/S0266462325102638

**Published:** 2025-12-29

**Authors:** Erica Aranha Suzumura, Annegret Hoch, Michael Urban

## Abstract

**Introduction:**

Cholesteatoma is an abnormal skin growth within the middle ear that can destroy the ossicular chain and cause hearing loss. This study aimed to evaluate the cost effectiveness of canal-wall-down (CWD) mastoidectomy for cholesteatoma removal with or without hearing reconstruction using an active transcutaneous bone conduction implant (BCI) in patients with advanced cholesteatoma to support the design of a future clinical study.

**Methods:**

A Markov model was developed to evaluate the cost effectiveness of the following strategies: CWD without hearing reconstruction (CWD only); CWD with simultaneous BCI implantation (CWD+BCI0); CWD with implantation of BCI after one year (CWD+BCI1), two years (CWD+BCI2), three years (CWD+BCI3), or five years (CWD+BCI5). The outcomes evaluated over a lifetime were quality-adjusted life years (QALYs), costs (from the perspective of the National Health Fund, Poland), and net monetary benefit (NMB), assuming a willingness-to-pay threshold of one gross domestic product (GDP) per capita in Poland (PLN92,955 [USD53,422]). One-way deterministic sensitivity analyses (DSA) were performed to evaluate the robustness of the results. Cost and NMB results were presented in 2024 PLN and USD (conversion using purchasing power parities for GDP).

**Results:**

The simulation indicated that CWD+BCI0 was expected to be the most effective strategy, yielding 18.35 QALYs, followed by CWD+BCI1, CWD+BCI2, CWD+BCI3, CWD+BCI5, and CWD only. The lifetime costs of performing CWD+BCI0 were expected to be lower than the costs of CWD+BCI1, CWD+BCI2, and CWD+BCI3. Moving from CWD only to CWD+BCI0 would incur an additional cost of PLN67,754 (USD38,939) over a lifetime to gain 3.13 QALYs. CWD+BCI0 resulted in the higher NMB (PLN1,595,530 [USD916,971]), followed by CWD+BCI1 (PLN1,578,976 [USD907,457]), CWD+BCI2 (PLN1,569,383 [USD901,944]), CWD+BCI3 (PLN1,560,090 [USD896,603]), CWD+BCI5 (PLN1,542,377 [USD886,424]), and CWD only (PLN1,372,572 [USD788,834]), respectively. DSA showed that the results were robust.

**Conclusions:**

CWD with simultaneous hearing reconstruction using a BCI (CWD+BCI0) is expected to be the most cost effective strategy for patients with cholesteatoma in the Polish context. This result will support the design of a clinical study evaluating the surgical and audiological outcomes of the simultaneous approach.

